# ‘Alone on our NF1 island’: a patient-led mixed-method survey study to understand the care pathway for neurofibromatosis type 1 (NF1) patients in the UK

**DOI:** 10.1136/bmjoq-2025-003383

**Published:** 2025-08-28

**Authors:** Shaowen Ju, Laura Cowley, Ishan Jain, Vanessa Martin, Ellie Day, Rona Smith, Tessa Morgan

**Affiliations:** 1 University of Cambridge, Cambridge, UK; 2 University of Cambridge Clinical School, Cambridge, UK; 3 Patient Led Research Hub, Cambridge, UK; 4 Warrington and Halton Teaching Hospitals NHS Foundation Trust, Warrington, UK; 5 Childhood Tumour Trust, East Sussex, UK; 6 Child Health, Royal Free London NHS Foundation Trust, London, UK; 7 Cambridge University Hospitals NHS Foundation Trust, Cambridge, UK

**Keywords:** Qualitative research, Patient Advocacy, Patient satisfaction, Health services research

## Abstract

**Background:**

Neurofibromatosis type 1 (NF1), a rare genetic disorder characterised by neurofibroma growth, affects approximately 25 000 individuals in the UK. Its wide range of clinical manifestations presents significant challenges in providing comprehensive care for patients. In agreement with National Health Service England’s Commissioners, Childhood Tumour Trust initiated a patient-led service evaluation to understand existing care pathways and identify factors influencing patient satisfaction.

**Methods:**

The study was coproduced with patient charities, clinicians and the Patient Led Research Hub. Online surveys were composed for patients, families, carers (PFCs) and healthcare professionals (HCPs) and disseminated through charity and healthcare networks. Structured features were analysed using descriptive statistics to review pathways and examine correlations with care satisfaction. Free-text responses were coded and analysed thematically to explore PFCs’ and HPCs’ experiences.

**Results:**

A total of 1083 PFC and 94 HCP responses were received from across the UK (783 and 49 were complete, respectively). Overall, 54% PFCs expressed dissatisfaction with NF1 care. While London had a significantly higher satisfaction rate (64%; p=0.01) than the national average, Scotland (30%, p=0.01) and Northern Ireland (16%, p=0.01) had significantly lower rates. Influencing factors included poor care coordination, long specialist wait times and insufficient signposting to charities. Regarding diagnosis and management, 46 HCP roles, 35 referral routes and 16 sources of management guidelines were identified, indicating a lack of clear pathways and care standardisation. Free-text data revealed additional challenges, including limited education and information for families, low NF1 awareness among professionals, inequitable access to specialists and a desire for holistic care.

**Conclusions:**

This evaluation revealed UK-wide dissatisfaction with NF1 care and a pressing need for system-level changes to improve regional disparities and care coordination, enhance patient education and HCP training and establish standardised pathways with a holistic model to enable high-quality equitable care for all NF1 patients.

**WHAT IS ALREADY KNOWN ON THIS TOPIC**

- Existing literature shows reduced quality of life and significant psychological burden associated with neurofibromatosis type 1 (NF1), with limited information on medical pathways and care satisfaction.

**WHAT THIS STUDY ADDS**

- This study revealed widespread dissatisfaction with NF1 care, care inequalities across regions, and a lack of standardised and integrated care pathways within the UK.

**HOW THIS STUDY MIGHT AFFECT RESEARCH, PRACTICE OR POLICY**

- Our findings highlight the need for policy changes to establish standardised, integrated care pathways, enhance awareness and education and improve access to holistic and equitable care for NF1 in the UK.

## Introduction

 Neurofibromatosis type 1 (NF1) is a genetic neurocutaneous disorder characterised by the development of neurofibromas (benign nerve sheath tumours), affecting an estimated 25 000 individuals within the UK.^1^ It is inherited in an autosomal dominant pattern with a birth incidence of 1 in 2500 to 1 in 3300.^2^ Individuals with NF1 have a reduced life expectancy of approximately 8–15 years less than the general population.^3 4^ Clinical manifestations and severity vary greatly, with complications across multiple systems, including the skin, bone and nervous system. Furthermore, learning and developmental difficulties are common among individuals affected, reflecting the importance of a multidisciplinary approach to care.^5^

In the UK, specialised care for NF1 is available at two nationally commissioned highly specialised services (HSSs): Manchester University Hospitals National Health Service (NHS) Foundation Trust and Guy’s and St Thomas’ NHS Foundation Trust in London. These centres provide comprehensive care for patients with rare and significant complications meeting specific criteria or ‘complex’ NF1.^6^ However, the vast majority of patients are managed outside these facilities where guidelines and recommendations are less well defined.^7^ Furthermore, research on the delivery and quality of care for this larger, ‘non-complex’ NF1 patient population remains limited, with most existing literature based on small sample sizes and without a specific focus on medical pathways.^8^,^11^ In 2021, the Department of Health and Social Care released the UK Rare Disease Framework, aiming to improve the lives of people living with rare diseases.^12^ However, despite a series of action plans published across each devolved nation, the NF1 care pathway remains disordered and ineffective.

To address this knowledge gap and evaluate the effectiveness of current health services, Childhood Tumour Trust (CTT), a NF1 charity supporting patients and families, initiated a service evaluation in agreement with NHS England’s HSS Commissioner. This project aims to explore patients, families and carers’ (PFCs’) and healthcare professionals (HCPs’) perspectives of strengths and areas of improvement of existing NF1 care pathways.

### Aim and objectives

The primary objective of this study is to investigate the perceptions and experiences of existing care pathways among PFCs living with NF1 in the UK.

Secondary objectives are to:

1. Identify the key factors underlying PFCs’ satisfaction and dissatisfaction with their care.
2. Compare and contrast PFCs’ and HCPs’ perspectives on NF1 care pathways.

## Methods

### Patient and public involvement

This study was led by CTT, a patient charity and supported by the Patient Led Research Hub, ensuring strong patient and caregiver representation.^13^ Two online surveys, one for PFCs and one for HCPs, were codeveloped with patient support groups (CTT, Tumour Support Scotland, Children’s Tumor Foundation (USA)), medical consultants experienced in NF1 care and a clinical trialist, to ensure questions were robust, relevant and aligned with patient families’ priorities and experiences. Conducted via Qualtrics, the surveys were disseminated through patient charities and other established networks, including professional associations (eg, British Paediatric Neurology Association, British Academy of Childhood Disability, British Association for Community Child Health), healthcare organisations and research teams. The study’s findings will be communicated back to the NF1 patient community through CTT-led dissemination strategies, including online reports and presentations at patient advocacy events.

### Survey design

Surveys comprised both structured and unstructured questions. Structured questions gathered non-identifiable demographic information, geographic location and details regarding care experiences. Unstructured questions invited open-ended responses focusing on suggestions for improvement and general experiences with the NF1 care pathway. Questions were bespoke but based on literature review to ensure findings could relate to published guidelines. The complete surveys are provided in online supplemental appendix A (PFC) and online supplemental appendix B (HCP).

The surveys were piloted with NF1 families from collaborating patient charities and medical consultants to ensure accessibility and relevance, with all feedback incorporated into the final version. They were available for completion for a period of 4 months (September to December 2022) and invited anonymous responses from individuals residing in the UK.

### Data analysis

A mixed-methods approach was employed: quantitative and qualitative analyses were conducted separately, and findings were integrated afterwards using the triangulation protocol to explore similarities and discrepancies.^14^ Structured features of the data were analysed using descriptive statistics. χ^2^ tests were used to explore associations between various factors and care satisfaction levels using categorical data. A heat map was generated to visually represent geographic distribution of satisfaction levels.

For qualitative analysis, free-text responses to the questions ‘What changes could be made to improve your/your child’s NF1 care?’ (PFC only), ‘What additional resources or support do you think are required for a Paediatric (HCP) and Adult (HCP) to coordinate holistic care for NF1 patients?’ (HCP only) and ‘Please add anything else you’d like to share regarding the current pathway of care for people with NF1 in the UK’. (PFC and HPC) were coded independently by two authors (SJ and IJ), both medical students who worked with NF1 families. These independent codes were discussed with a senior qualitative researcher (TM) to iteratively explore similarities and discrepancies to support the consistency and reliability of the findings. Two researchers (SJ and IJ) clustered and categorised codes to identify potential themes in line with principles of thematic analysis.^15^ To deepen and refine our analysis, we collaboratively compared and contrasted PFC and HCP responses. The identified themes were reviewed and finalised with two additional authors (TM and LC) and iteratively discussed with the charity partner (author VM) to ensure the trustworthiness of our findings from the patient perspective. This multitiered discussion led to the final consolidation of the themes. Both positive and negative cases were included to ensure the analysis reflected the diversity of respondents’ views and transparency of our findings.^16^

## Results

### Respondent characteristics

A total of 1083 PFC and 94 HCP responses were received, of which 784 and 49 were, respectively, completed in full. Only completed responses were included in the analyses. PFC responses encompassed all age ranges, with 76% representing a patient 20 years or younger. The majority (77%) of PFCs were parents or carers, while 23% were patients.

Among HCP respondents, 88% provided care for someone with NF1: 49%, 19%, 12% and 21% had 1–10, 11–20, 21–30 or over 30 patients, respectively. They had diverse roles, the majority of which were paediatricians (41%), general practitioners (GPs) (18%) and nurses (14%). 65% of HCPs provided paediatric healthcare.

Regarding geographical location, PFC respondents received NF1 care in every UK nation and England region, while HCP respondents provided care in Northern Ireland, Scotland and each region of England. The distribution by number and percentage from each region is presented in table 1.

**Table 1:** Number and percentage of patients, families and carers (PFCs) and healthcare professionals (HCPs) by region

Region	PFC	HCP
East of England	47 (6%)	3 (6%)
Northeast and Yorkshire	79 (10%)	9 (18%)
Northern Ireland	19 (2%)	1 (2%)
North West	93 (12%)	2 (4%)
Scotland	69 (9%)	6 (12%)
South East	146 (19%)	8 (16%)
South West	101 (13%)	9 (18%)
Wales	56 (7%)	0 (0%)
Midlands	130 (17%)	3 (6%)
London	44 (6%)	8 (16%)

### Quantitative analyses

#### Factors influencing care satisfaction

**Figure 1:**
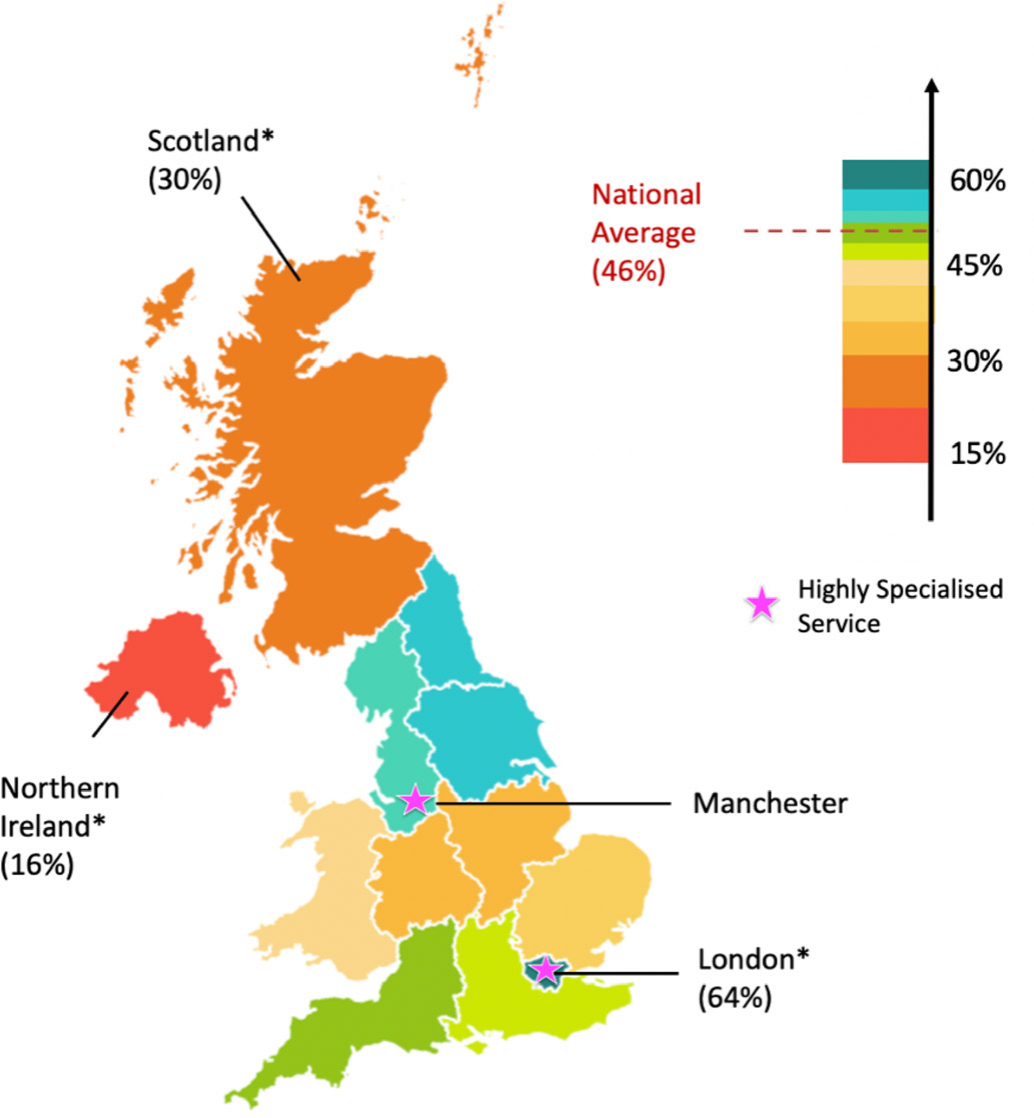
Heat map of regional satisfaction rates. London (64%, p=0.01), Northeast and Yorkshire (53%), Northwest (52%), Southwest (51%), Southeast (48%), Wales (43%), East England (40%), Midlands (40%), Scotland (30%, p=0.01) and Northern Ireland (16%, p=0.01); *Statistical significance relative to the national average; pink stars indicate regions where specialist centres for neurofibromatosis type 1 (NF1) are located.

Overall, 54% of PFCs expressed dissatisfaction with NF1 care, with significantly lower satisfaction in Scotland (30%, p=0.01) and Northern Ireland (16%, p=0.01) and higher in London (64%; p=0.01) (figure 1) when compared with the national average. Potential influencing factors included poor care coordination, long specialist wait times and insufficient signposting to charities. Specifically, PFCs without a care coordinator were significantly less satisfied (25%; p<0.001) than those with one (71%; p<0.001), highlighting the importance of care coordination in satisfaction. In addition, only 42% PFCs saw a specialist within 6 months of diagnosis, indicating long specialist wait times for the majority of patients. Even fewer PFCs (30%) were signposted to charities, with referral rates significantly lower in East England (15%; p=0.02) and Northern Ireland (5%; p=0.02), suggesting the potential impact of charity support on care satisfaction.

#### Pathways for referral, diagnosis and management

The referral process for NF1 was highly variable, with 35 routes identified by PFCs and HCPs, and 30% of HCPs stating they were unaware of how to contact the HSS for advice or patient referral. Furthermore, PFCs and HCPs identified 46 different professional roles involved in diagnosing NF1, evidencing the range of expertise required and the need for multidisciplinary input in NF1 care provision. Similarly, care pathways postdiagnosis were unclear, with 63% of PFCs stating it was not obvious after diagnosis who would be providing care. Moreover, NF1 management differed between providers. Despite 72% of HCPs reporting using guidelines, 16 different guidelines were cited, demonstrating a lack of standardisation in NF1 management.

#### Pathway accessibility and care integration

Despite numerous pathways, accessibility issues were frequently reported. In between scheduled appointments, although 98% of HCPs claimed availability for contact as needed, 45% of PFCs reported difficulties in accessing HCPs with NF1 expertise. Poor care integration and coordination also made key services hard to access. For example, only 45% of PFCs reported having a designated key healthcare provider and 41% access to a specialist advisor nurse. The deficiency in holistic care was also reported by HCPs. While 72% of HCPs felt responsible for coordinating holistic care for their patients, 69% believed that the key providers and care coordinators did not have enough knowledge and support (including financial) necessary for their work.

### Qualitative analyses

#### Limited education and information for families

PFCs expressed significant concerns regarding the lack of information and educational resources for NF1, particularly those provided by the NHS. In some cases, this deficiency has contributed to the misinterpretation of medical guidance. For example, despite guidelines suggesting the limited utility of MRI for most NF1 cases,^7^ many PFCs advocated for baseline MRIs for all individuals with NF1, viewing them as essential for ongoing disease monitoring.

In addition to disease-related information, many PFCs lacked knowledge about care pathways and expectations of their care. This knowledge gap prompted many to seek guidance from charities and support groups outside the NHS. A representative sentiment was expressed as: “*It all feels ad hoc. Clear info on what to expect, who to contact etc would be beneficial. The best info came from CTT [Childhood Tumour Trust]—the red book insert [a page of NF1-related information made to fit in the UK child health record]*.”

Furthermore, the difficulties in accessing information and guidance were exacerbated by the absence of a centralised first point of contact. This left PFCs feeling disoriented and isolated in their NF1 journey, as one parent lamented: “*It is mystifying who [our baby] is meant to see and when. There is not much information or guidance or help. We are pretty much alone on our NF1 island*.”

In conclusion, PFCs reported inadequate patient education and availability of NF1-related information through the NHS. There appeared to be a pressing need for centralised, clear and accessible educational resources to empower and support PFCs effectively.

#### Professionals lacking awareness

Besides the lack of knowledge among PFCs, a lack of familiarity with NF1 was also reported among HCPs, especially among GPs. Despite the crucial role GPs play in the care of NF1 patients, HCPs reported that GP practices often lacked the staffing, funding and ‘access to clear guidance and advice’ necessary to provide adequate care. From PFCs’ perspective, this lack of NF1 awareness eroded their confidence in the medical profession and resulted in significant diagnostic delays, as one respondent recounted, *“It took asking 12 different GPs in 12 years about what I now know to be neurofibromas before I was finally referred to a dermatologist.”*

Moreover, HCPs’ lack of awareness was sometimes perceived as a dismissive attitude, as reflected in the comment: *“Schools and healthcare professionals must know the basics! Parents must be heard!”* Similarly, HCPs also recognised their own gaps in knowledge and advocated for enhanced training opportunities, including “*training via hands-on experience*,” “*awareness training for all healthcare staff*,” “*access to specialist training*,” and “*educational opportunities and support networks*.” These requests from HCPs echoed with PFCs’ experiences and highlighted the need for improving NF1 awareness and training across the healthcare system.

#### Regional disparities

Besides the lack of education for both PFCs and HCPs, regional differences in guidelines, care quality and access to specialist services also contributed to dissatisfaction across the UK. In general, respondents referred to the specialist centres as ‘superior’ and ‘excellent’, while expressing disappointment in NF1 services outside these centres, particularly in Northern Ireland and Scotland. For example, some respondents described the services in Northern Ireland as ‘diabolical’ and ‘just not good enough’. In addition, the services in Scotland were also seen as inferior compared with those in England, as one parent summarised: “*My son was born in England and he had access to NF1 clinic and had good care… In Scotland we have no NF1 clinic for us to attend, we have to fight for appointments… I can’t understand why the care is so different in areas every child deserves the same great care*”.

This geographical inequality has led to what PFCs described as a ‘postcode lottery’, with access to specialist care concentrated in London and Manchester, driven by expertise within the HSSs. This uneven distribution of NF1 specialists was seen as an important cause of the care inequality, as exemplified by the statement: “*It’s a postcode lottery people living near the centres get referred as it’s their local team despite their need not being as high as thousands of others who never get to see anyone who even has an interest in NF1*”. Similarly, this geographic disparity in specialist access was also reported by HCPs, who demanded ‘localised specialist centres and specialists available within regions across the UK’.

Limitation in financial resources was identified as another cause of regional disparities, as evidenced by the call for increased staffing and funding for services and research from both PFCs and HCPs. However, despite these issues, some respondents reported positive experiences, with one exclaiming, “*I have ALWAYS HAD FANTASTIC CARE from all the different hospitals I've been in*”.

#### Disconnected care

PFCs expressed dissatisfaction with the disjointed nature of NF1 care. Responses evidenced that effective NF1 management requires a cohesive, multidisciplinary approach; however, PFCs reported a significant lack of coordination among HCPs. This fragmented system led some PFCs to take on care management responsibilities themselves, as encapsulated in the statement: *“It’s not coordinated at all and really needs to be—there are no specialists (apart from London/Manchester) and no one wants to take responsibility or make it a priority.”*

Moreover, PFCs found the care pathways confusing and ineffective, describing them as ‘unclear’, ‘rubbish’ and ‘not fit for purpose’. The inadequacy of these pathways prompted desperate pleas from PFCs, such as “Can we please, please, please, please have a NF1 Pathway of Care?” Furthermore, this sentiment was shared by HCPs, who called for ‘clear pathways of care’, ‘clear national guidelines’ and improved ‘pathway accessibility’.

From PFCs’ perspective, the absence of clearly defined care pathways often led to delays in detection and diagnosis, extended waiting times and insufficient routine assessments and follow-ups. In addition, many PFCs reported the gap between paediatric and adult services, as one parent remarked: “*There is no care once your child is 18*.” Similarly, this lack of transition services was highlighted by HCPs, as exemplified by the comment: “*age transitions are patchy or non-existent unless part of a specialist service*”.

Collectively, both PFCs and HCPs advocated for the development of effective age-inclusive care pathways that ensured coordinated care across multiple disciplines.

#### Demand for holistic care

Due to the lack of integrated pathways, holistic care remained inaccessible to many PFCs. Given NF1’s broad clinical spectrum, many patients required comprehensive support that extended beyond physical health to encompass emotional, behavioural and educational dimensions, which were not sufficiently addressed by current pathways. A PFC described this gap as: “*… the current pathway of care is very much focused on the physical impact/symptoms of NF1, not the learning/behavioral/mental health side… There is no joining up of these aspects of the condition”*.

Educational support was identified as a critical area needing improvement, particularly in providing age-appropriate support for adolescents. PFCs reported that NF1 was often not recognised as a disability in educational settings, making it difficult to access support for associated learning difficulties and behavioural issues. One PFC explained the issue as: “*…the behavior side—ADHD [Attention Deficit Hyperactivity Disorder] and Autism—how NF kids can be affected. More help and advice from schools to support our children*”.

Furthermore, PFCs described a significant lack of emotional support, especially at the time of diagnosis, as one respondent stated: “*Emotional support is vital at the start of diagnosis as it can feel incredibly overwhelming for the family*”. In contrast, HCPs raised fewer concerns about access to mental health and educational support. Instead, they highlighted the need for ‘liaison with NF1 specialist nurses’, suggesting the differences in perceived and actual priorities of PFCs.

Overall, while the current care pathway tended to focus predominantly on physical symptoms, it frequently overlooked the emotional, behavioural and educational needs of patients. There was a unanimous call among HCPs and PFCs for an integrated care pathway with a holistic model of care for NF1.

## Discussion

### Need for standardisation and clear pathways

This study represents the first patient-led service evaluation of NF1 healthcare across the UK. Despite there being multiple guidelines around the provision of NF1 care,^5 7 17^ drawing on evidence from families, carers and HCPs across the UK, we identified a definite lack of standardisation in NF1 care provision. Using a mixed-methods approach that compared and contrasted PFCs’ and HCPs’ perspectives, our findings revealed nationwide dissatisfaction among PFCs, with varying satisfaction levels across regions. While London reported significantly higher levels of satisfaction compared with the national average, Scotland and Northern Ireland had significantly lower levels.

The quantitative findings reveal several contributing factors to these disparities, including unequitable access to specialist services, inadequate signposting to charities and delays in diagnosis and referral. Qualitative data further highlight additional challenges, such as (1) limited NF1 education and information for both PFCs and HCPs; (2) poor interprofessional and longitudinal care coordination, especially during the transition from paediatric to adult services and (3) a lack of holistic care, especially in addressing mental, behavioural and educational needs. These findings emphasised the need for establishing standardised care protocols and clear, effective care pathways—both regionally and across professional disciplines—particularly to improve care for patients managed outside of the HSSs.

### Relation to the UK rare disease framework

Identified areas for improvement directly align with the key priorities in the UK Rare Diseases Framework: (1) enabling faster diagnosis, (2) increasing awareness among HCPs, (3) improving care coordination and (4) improving access to specialist care and treatments.^12^ Furthermore, our findings highlight particular deficiencies in priorities 2 and 3, which are the least funded across all rare disease research in the UK, receiving 7.1% and 4.3% of total grants, respectively.^18^ Most of these awards focused on at least one other priority, suggesting the proportion of research dedicated to improving awareness or care coordination was minimal.

Moreover, our findings on geographical disparities mirror trends observed across the UK. The greatest proportion of rare disease research funding was concentrated in Greater London (39%), while Scotland, despite ranking third in total funded projects (11.4%),^18^ showed significantly lower satisfaction levels for NF1 care compared with the national average. This disparity suggests that NF1 may be underfunded relative to other rare diseases in Scotland. Further investigation is warranted to understand the distribution of NF1-specific funding and broader inequities in research support across both the Framework priorities and geographical regions in the UK.

### Recommendations

This study identified four key ways for improvement: (1) establishing standardised care pathways, (2) improving awareness and education for PFCs and HCPs, (3) enhancing care coordination and (4) promoting access to holistic, multidisciplinary care. Patient charities have played a crucial role in addressing some of these gaps. For example, CTT provides NF1-specific training for HCPs through Continuing Professional Development courses,^19^ while Nerve Tumours UK supports NF Specialist Advisor nurses who help coordinate care.^20^ Despite these efforts, systemic change at the national level is essential to ensure accessible and equitable care for all NF1 patients. In developing guidelines and pathways, it is critical that the Framework priorities are explicitly addressed, with particular focus on improving awareness and care coordination, including improving the transition from paediatric to adult services.

NF1 presents distinct challenges compared with other rare conditions. First, it has a highly variable clinical spectrum, meaning patients have diverse and evolving needs. In the UK, patients are categorised as having either ‘complex’ or ‘non-complex’ NF1, with care provision differing markedly between these groups. Currently, the absence of clear care pathways and standardised protocols disproportionately affects the non-complex population. Second, clinical manifestations can emerge or progress over time, meaning that patients initially classified as non-complex may later require complex care. As such, national guidelines should include standardised monitoring protocols for non-complex patients to ensure early detection of complications and timely intervention.

Additionally, because NF1 is more common than many other rare diseases, it is impractical to centralise care for all patients within specialised centres. Instead, a tiered ‘hub-and-spoke’ model, such as that used for paediatric epilepsy, could be adapted for NF1.^21^ In this model, patients receive care from local paediatricians with additional expertise in epilepsy at the secondary care level, while those with complex needs are referred to tertiary-level paediatric neurologists.^21^ This framework could provide a scalable and sustainable solution for NF1, ensuring that patients across the complexity spectrum receive appropriate care.

### Strengths and limitations

The strengths of this study are in its patient-driven, multistakeholder approach, with strong interest from PFCs and HCPs reflected by the large number of detailed responses for a rare disease. Furthermore, combining qualitative with quantitative analysis, this study allowed the breadth and variety of the response to be explored, as well as contextualising and exploring the trends identified in the quantitative analysis on a deeper level.

Additionally, this study is the first patient-led service evaluation of NF1 healthcare service in the UK, demonstrating the value of patient and public partnership in health research. Comparing and contrasting PFCs’ and HCPs’ views, this service evaluation attempted to provide a balanced view of the experiences with the healthcare services among NF1 patients and healthcare providers in the UK.

This evaluation was limited by its non-random sampling method and the use of potentially ambiguous terminologies, like ‘NF1 specialists’, in survey questions. In addition, there was an uneven split between the number of PFC and HPC responses, which biased the results towards PFCs’ perspective. Because care satisfaction and pathway analyses are sparse in literature, it is difficult to compare our results to other rare diseases.

Nevertheless, this study offered valuable insights into current challenges within the NF1 care pathway and represented a first step towards identifying key areas for future research and development. Future research should further investigate the specific mechanisms driving regional disparities and inconsistencies in NF1 care, particularly focusing on the ‘non-complex’ patient population. Detailed analyses of local service models, referral pathways and transitions from paediatric to adult services will be crucial to identify structural gaps and inform targeted interventions.

## Conclusions

In conclusion, this evaluation revealed UK-wide dissatisfaction with the NF1 care model and regional disparities. It advocates for policy changes to address care inequalities, the development of standardised guidelines and pathways, improvements to HCP training and patient education and enhanced access to coordinated and holistic care for all patients.

## Supplementary material

10.1136/bmjoq-2025-003383 online supplemental file 1

10.1136/bmjoq-2025-003383 online supplemental file 2

## Data Availability

Data are available on reasonable request.
