# Supplementary material for: ‘Alone on our NF1 island’: a patient-led mixed-method survey study to understand the care pathway for neurofibromatosis type 1 (NF1) patients in the UK
Source: BMJ Open Qual. 2025 Aug 28;14(3):e003383. doi: 10.1136/bmjoq-2025-003383 (PMC12410633; doi:10.1136/bmjoq-2025-003383)
Supplement: online supplemental file 1 [file bmjoq-14-3-s001.docx]

Patient, Family, Carer Survey

Start of Block: intro

Introduction
**Neurofibromatosis Type 1 (NF1): understanding the care pathway** 
**Anonymous survey for people affected by NF1 in the UK**
  
As part of our commitment to improving care for patients, particularly in local areas, and following agreement with the commissioners of the Complex NF1 Service, Childhood Tumour Trust is initiating research into how people with NF1 can be supported outside of the Complex Service. As a first step, the charity has been working with the Patient Led Research Hub (plrh.org) to learn more about the existing care pathway. 


If you, a family member, or someone you care for has been diagnosed with NF1 please consider completing this survey. Responses are welcome from anyone living in the UK (you do not need to be a Complex Service patient); you can complete the survey multiple times for different individuals, if needed. The survey has been designed by Childhood Tumour Trust and externally reviewed by NF1 charities and healthcare professionals. The responses will be analysed to highlight potential areas of need that we can explore with future research projects. This survey is **anonymous** and should take **less than 10 minutes to complete**. 

If you are a healthcare provider, please consider sharing your professional NF1 experience through our Healthcare Professionals Survey. You are welcome to share both survey links with anyone affected by, or providing healthcare for, NF1.  

Thank you for your interest in this project. If you would like help completing this survey, or if you have any questions or feedback, please contact Laura Cowley (Research Lead for the Patient Led Research Hub: lbm28@cam.ac.uk).

End of Block: intro

Start of Block: Question Block

Are you completing this survey as a:

- Person diagnosed with NF1
- Parent or carer to someone diagnosed with NF1

Explanation “Child” For ease of reading, we use the words ‘your child’ throughout this survey. However, we recognise that you may be their carer and not parent, or that your child may be now be an adult.

| Page Break |  |
| --- | --- |

In what region are you/your child regularly monitored for NF1?

- East of England
- London
- Midlands
- North East & Yorkshire
- North West
- South East
- South West
- Scotland
- Wales
- Northern Ireland

| Page Break |  |
| --- | --- |

What is your/your child's current age?

- Under 1 year
- 1-3 years
- 4-8 years
- 9-13 years
- 14-20 years
- Older than 20 years

| Page Break |  |
| --- | --- |

What age did you (or your family or friends) first notice signs or symptoms that you now realise are related to NF1?

- Under 1 year
- 1-3 years
- 4-8 years
- 9-13 years
- 14-20 years
- Older than 20 years

| Page Break |  |
| --- | --- |

What age were NF1 signs or symptoms first noticed or acknowledged by a healthcare professional?

- Under 1 year
- 1-3 years
- 4-8 years
- 9-13 years
- 14-20 years
- Older than 20 years

| Page Break |  |
| --- | --- |

What was the **first** sign or symptom noticed?

|  | By patient, family or carer | By a healthcare professional |
| --- | --- | --- |
| Café au Lait spots |  |  |
| Freckling (armpit or groin) |  |  |
| Lump |  |  |
| Bone problems |  |  |
| Eye changes |  |  |
| Difficulties in developmental milestones |  |  |
| Difficulties in education |  |  |
| Other (please specify) |  |  |

| Page Break |  |
| --- | --- |

What healthcare professional first noticed or acknowledged these signs or symptoms?

- General Practitioner (family GP)
- Health Visitor
- Paediatrician
- Ophthalmologist (eye doctor)
- Dermatologist (skin specialist)
- Neurologist
- Geneticist
- NF Specialist Advisor / NF Specialist Nurse
- They were not noticed until I raised my concerns
- Other (please specify) __________________________________________________

| Page Break |  |
| --- | --- |

After the signs or symptoms were noticed, how long did you/your child wait before seeing a healthcare professional with expertise in NF1?

- 0-6 months
- 7-11 months
- 1-2 years
- More than 2 years
- Other (please specify) __________________________________________________
- Have not yet seen someone with NF1 expertise

| Page Break |  |
| --- | --- |

Who referred you/your child to a healthcare professional with expertise in NF1?

- General Practitioner (family GP)
- Health Visitor
- Paediatrician
- Ophthalmologist (eye doctor)
- Dermatologist (skin specialist)
- Neurologist
- Geneticist
- Nerve Tumour UK helpline
- Unsure
- Have not yet been referred
- Other (please specify) __________________________________________________

| Page Break |  |
| --- | --- |

What age were you/your child formally diagnosed with NF1?

- Under 1 year
- 1-3 years
- 4-8 years
- 9-13 years
- 14-20 years
- Older than 20 years
- Have not yet received a formal diagnosis

| Page Break |  |
| --- | --- |

Who made the formal diagnosis for you/your child?

- General Practitioner (family GP)
- Paediatrician
- Ophthalmologist (eye doctor)
- Dermatologist (skin specialist)
- Neurologist
- Geneticist
- Other (please specify) __________________________________________________
- Not applicable (not formally diagnosed)

| Page Break |  |
| --- | --- |

Is there a history of NF1 in your family?

- Yes: it was inherited from a parent
- No: it is the first case that we know of
- Unsure

| Page Break |  |
| --- | --- |

Was your/your child’s diagnosis confirmed with genetic testing?

- Yes
- No
- Unsure

| Page Break |  |
| --- | --- |

Was it obvious to you, after suspected or confirmed diagnosis, who would be providing NF1 care for you/your child? (i.e. was there a clear ‘pathway of care’?)

- Yes
- No

| Page Break |  |
| --- | --- |

Do you have a key healthcare professional who organises all of your/your child’s NF1 care?

- Yes
- No

| Page Break |  |
| --- | --- |

Display This Question:

If Do you have a key healthcare professional who organises all of your/your child’s NF1 care? = Yes

When under the age of 18 years (**paediatric patient**), who is/was responsible for organising you/your child’s NF1 care?

- General Practitioner (family GP)
- Health Visitor
- Paediatrician
- Paediatric Neurologist
- Neurologist
- Ophthalmologist (eye doctor)
- Dermatologist (skin doctor)
- Geneticist
- Community Care Nurse
- Specialist Nurse
- Other (please specify) __________________________________________________

| Page Break |  |
| --- | --- |

Display This Question:

If Do you have a key healthcare professional who organises all of your/your child’s NF1 care? = Yes

When 18 years or older (**adult patient**), who is responsible for organising you/your child’s NF1 care?

- General Practitioner (family GP)
- Neurologist
- Ophthalmologist (eye doctor)
- Dermatologist (skin doctor)
- Geneticist
- Community Care Nurse
- Specialist Nurse
- Other (please specify) __________________________________________________
- Not applicable (under the age of 18 years)

| Page Break |  |
| --- | --- |

Display This Question:

If Do you have a key healthcare professional who organises all of your/your child’s NF1 care? = No

Who do you think **should** be responsible for organising NF1 care for children under the age of 18 years (**paediatric patients**)?

- General Practitioner (family GP)
- Health Visitor
- Paediatrician
- Paediatric Neurologist
- Neurologist
- Ophthalmologist (eye doctor)
- Dermatologist (skin doctor)
- Geneticist
- Community Care Nurse
- Specialist Nurse
- Other (please specify) __________________________________________________

| Page Break |  |
| --- | --- |

Display This Question:

If Do you have a key healthcare professional who organises all of your/your child’s NF1 care? = No

Who do you think **should** be responsible for organising NF1 care for people 18 years or older (**adult patients**)?

- General Practitioner (family GP)
- Neurologist
- Ophthalmologist (eye doctor)
- Dermatologist (skin doctor)
- Geneticist
- Community Care Nurse
- Specialist Nurse
- Other (please specify) __________________________________________________

| Page Break |  |
| --- | --- |

Were you given the opportunity to discuss the possible complications that can be associated with NF1, what to look for, and who to contact if needed?

- Yes
- No

| Page Break |  |
| --- | --- |

Were you introduced to patient support groups and/or the national patient charities (Childhood Tumour Trust, Nerve Tumours UK, or Tumour Support Scotland)?

- Yes
- No

| Page Break |  |
| --- | --- |

Are you/your child seen in a clinic with an NF Specialist Advisor (sometimes called an NF Specialist Nurse)?

- Yes
- No
- Unsure

| Page Break |  |
| --- | --- |

How often do you/your child see a healthcare professional with expertise in NF1?

- At least once per year
- Annually
- Once every 2 years
- Only as needed
- Other (please specify) __________________________________________________

| Page Break |  |
| --- | --- |

Are you able to contact a healthcare professional with NF1 expertise as needed, in between scheduled appointments?

- Yes
- No

| Page Break |  |
| --- | --- |

What measurements are recorded **each time** you/your child has an NF1 check-up? Tick all that apply.

- Head circumference
- Height
- Weight
- Blood pressure
- Other (please specify) __________________________________________________
- ⊗Unsure

| Page Break |  |
| --- | --- |

What assessments are completed **at least once a year** as part of your/your child’s NF1 care? Tick all that apply.

- Skin
- Spine and skeleton
- Eyes (yearly until the age of 8; may be less frequent after that)
- Heart and lungs
- Brain (headaches, balance, etc)
- Puberty
- Education and behaviour
- Other (please specify) __________________________________________________
- ⊗Unsure

| Page Break |  |
| --- | --- |

Was the Highly Specialised Complex NF1 Service and its eligibility criteria explained to you (e.g. why only some patients are referred)? The Service is provided by Manchester University Hospitals NHS Foundation Trust, and Guy’s and St Thomas’ NHS Foundation Trust.

- Yes
- No

| Page Break |  |
| --- | --- |

Have you/your child ever been referred to the Highly Specialised Complex NF1 Service provided by Manchester or Guy’s or St Thomas’ hospitals?

- Yes
- No

| Page Break |  |
| --- | --- |

Display This Question:

If Have you/your child ever been referred to the Highly Specialised Complex NF1 Service provided by... = Yes

How often are you/your child seen at the Complex NF1 Specialist Service?

- At least once per year
- Annually
- Every 2 years
- As requested
- Seen once and then discharged
- Other (please specify) __________________________________________________

| Page Break |  |
| --- | --- |

Please indicate which, if any, services you or your child have been referred to:

|  | Yes | No | Not applicable |
| --- | --- | --- | --- |
| Eye doctor |  |  |  |
| Child and adolescent mental health |  |  |  |
| Adult mental health |  |  |  |
| Educational support |  |  |  |
| Breast cancer screening (directly or via GP) |  |  |  |
| Genetic counselling (including family planning) |  |  |  |
| Speech & language therapy |  |  |  |
| Occupational therapy |  |  |  |
| Physiotherapy |  |  |  |

| Page Break |  |
| --- | --- |

Please tick any of the below that you or your child have experienced:

- Plexiform neurofibromas
- Optic glioma
- Pseudarthrosis
- Scoliosis
- Attention Deficit Hyperactivity Disorder (ADHD)
- Autistic Spectrum Disorder (ASD)
- Learning difficulties
- Hypermobility
- NF1-related internal tumours (e.g. on the spine, bowel, etc)
- Other NF1 complications (please specify) __________________________________________________
- ⊗None of the above

| Page Break |  |
| --- | --- |

Do you feel you/your child have non-NF1 health issues which complicate NF1 care or management?

- Yes
- No
- Unsure if other health issues are related to NF1

Skip To: mgmt If Do you feel you/your child have non-NF1 health issues which complicate NF1 care or management? = No

| Page Break |  |
| --- | --- |

non-NF1 care Please explain how you think other health issues impact your/your child's NF1 care or management. **Please do not provide any personal or confidential information.**

________________________________________________________________

________________________________________________________________

________________________________________________________________

________________________________________________________________

________________________________________________________________

| Page Break |  |
| --- | --- |

In your opinion, what are the most important strategies for managing NF1?

|  | Not important | Somewhat important | Very important |
| --- | --- | --- | --- |
| Understanding diagnosis and clinical information |  |  |  |
| Diagnosis of learning and behavioural disorders |  |  |  |
| Support for learning and behavioural difficulties |  |  |  |
| Support around emotional wellbeing and mental health |  |  |  |
| Support for education |  |  |  |
| Access to early screening for physical health (e.g. scans) |  |  |  |
| Direct access to NF1 specialist |  |  |  |

| Page Break |  |
| --- | --- |

How was your/your child’s NF1 care managed when moving from paediatric to adult services?

- Transition clinic offered with both paediatric & adult care team in attendance
- Counselled on change in services and new care pathway explained
- Referred to specialist NF1 adult services
- Referred to non-NF1 adult services
- Discharged to family GP
- My child is still under paediatric care
- I/my child did not attend paediatric services
- Other (please specify) __________________________________________________

| Page Break |  |
| --- | --- |

Are you satisfied with the way your/your child’s NF1 care is managed?

- Yes
- No

| Page Break |  |
| --- | --- |

Display This Question:

If Are you satisfied with the way your/your child’s NF1 care is managed? = No

What changes could be made to improve your/your child's NF1 care?

________________________________________________________________

________________________________________________________________

________________________________________________________________

________________________________________________________________

________________________________________________________________

| Page Break |  |
| --- | --- |

| 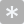 |
| --- |

In your opinion, what are the top 3 research priorities for NF1?

- Benefits of full body baseline scan
- Special educational needs
- Correlation with Autism Spectrum Disorder and/or Attention Deficit Hyperactivity Disorder
- Hypermobility
- Pain management
- Management of plexiform neurofibromas
- Relief from itching
- Bowel issues
- Monitoring for malignancy
- Treatment of cutaneous neurofibromas
- Other (please specify) __________________________________________________

End of Block: Question Block

Start of Block: feedback

Please add anything else you’d like to share regarding the current pathway of care for people with NF1 in the UK.

________________________________________________________________

________________________________________________________________

________________________________________________________________

________________________________________________________________

________________________________________________________________

End of Block: feedback
