# Supplementary material for: ‘Alone on our NF1 island’: a patient-led mixed-method survey study to understand the care pathway for neurofibromatosis type 1 (NF1) patients in the UK
Source: BMJ Open Qual. 2025 Aug 28;14(3):e003383. doi: 10.1136/bmjoq-2025-003383 (PMC12410633; doi:10.1136/bmjoq-2025-003383)
Supplement: online supplemental file 2 [file bmjoq-14-3-s002.docx]

Healthcare Professionals Survey

Start of Block: Intro

Introduction
**Neurofibromatosis Type 1 (NF1): understanding the care pathway** 
**Anonymous survey for healthcare professionals in the UK**
  
As part of our commitment to improving care for patients, particularly in local areas and following agreement with the commissioners of the Complex NF1 Service, Childhood Tumour Trust is initiating research into how people with NF1 can be supported outside of the Complex Service. As a first step, the charity has been working with the Patient Led Research Hub (plrh.org) to learn more about the existing care pathway.


If you are involved with, or interested in, providing NF1 care in the UK, please consider completing this survey. It's been designed by Childhood Tumour Trust and externally reviewed by NF1 charities and healthcare professionals. This survey forms part of a public consultation which may highlight areas of unmet need that can be explored with subsequent research. This survey is **anonymous** and should take about **5 minutes to complete**.

If you or a family member have been diagnosed with NF1, please consider sharing your personal experience through our Patient, Family and Carer Survey. You are welcome to share the survey links with your patients and/or colleagues.

Thank you for your interest in this project. If you have any questions or feedback, please contact Laura Cowley (Research Lead for the Patient Led Research Hub: lbm28@cam.ac.uk).

End of Block: Intro

Start of Block: Question Block

Are you completing this survey as a:

- Dermatologist
- General Practitioner
- Geneticist
- Health Visitor
- Neurologist
- Nurse - Community Care
- Nurse - Hospital
- Nurse - Specialist
- Ophthalmologist
- Paediatrician - Community
- Paediatrician - General
- Paediatrician - Specialist
- Paediatric Neurologist
- Other (please specify) __________________________________________________

| Page Break |  |
| --- | --- |

How are you involved in NF1? Tick all that apply.

- Clinical practice
- Academic / research interest
- Personal / family interest
- Other (please specify) __________________________________________________

| Page Break |  |
| --- | --- |

What region/s do you work in? Tick all that apply.

- East of England
- London
- Midlands
- North East & Yorkshire
- North West
- South East
- South West
- Scotland
- Wales
- Northern Ireland

| Page Break |  |
| --- | --- |

Have you provided care for someone with NF1?

- Yes
- No

Skip To: paed holistic care If Have you provided care for someone with NF1? = No

| Page Break |  |
| --- | --- |

Are your NF1 patients:

- Children (0-18yrs)
- Adults (older than 18yrs)
- Both

| Page Break |  |
| --- | --- |

How many patients with NF1 are **currently** under your care?

- 0
- 1-10
- 11-20
- 21-30
- Over 30

| Page Break |  |
| --- | --- |

Do you typically see patients before an official NF1 diagnosis has been made?

- Yes
- No

| Page Break |  |
| --- | --- |

Have you been involved in making a clinical NF1 diagnosis?

- Yes
- No

| Page Break |  |
| --- | --- |

If you provide NF1 care, who do you receive referrals from? Tick all that apply.

- Dermatologist
- General Practitioner
- Geneticist
- Health Visitor
- Nerve Tumours UK helpline
- Neurologist
- Nurse – Community Care
- Nurse – Hospital
- Nurse – Specialist
- Oncologist
- Ophthalmologist
- Orthopaedics
- Paediatrician – Community
- Paediatrician – General
- Paediatrician – Specialist
- Paediatric Neurologist
- Other (please specify) __________________________________________________
- ⊗Not applicable

| Page Break |  |
| --- | --- |

What features do you look for to indicate NF1? Tick all that apply.

- Café au Lait spots
- Inguinal and axillary freckling
- Neurofibromas
- Bone deformities
- Eye changes
- Difficulties in developmental milestones
- Difficulties in education
- Family history
- Other (please specify) __________________________________________________

| Page Break |  |
| --- | --- |

To your knowledge, what is the average age of your patients when diagnosed with NF1?

- Under 1 year
- 1-3 years
- 4-8 years
- 9-13 years
- 14-20 years
- Older than 20 years
- Unsure

| Page Break |  |
| --- | --- |

Do you complete any baseline investigations at NF1 diagnosis or first assessment (e.g. MRI)?

- Yes
- No

| Page Break |  |
| --- | --- |

Display This Question:

If Do you complete any baseline investigations at NF1 diagnosis or first assessment (e.g. MRI)? = Yes

Please list your baseline investigations.

________________________________________________________________

________________________________________________________________

________________________________________________________________

________________________________________________________________

________________________________________________________________

| Page Break |  |
| --- | --- |

Do you routinely follow any guidelines for the management of patients with NF1?

- Yes
- No

| Page Break |  |
| --- | --- |

Display This Question:

If Do you routinely follow any guidelines for the management of patients with NF1? = Yes

Please list the management guidelines you use.

________________________________________________________________

________________________________________________________________

________________________________________________________________

________________________________________________________________

________________________________________________________________

| Page Break |  |
| --- | --- |

How often do you routinely see each patient with NF1?

- At least once per year
- Annually
- Once every 2 years
- Only as needed
- Other (please specify) __________________________________________________

| Page Break |  |
| --- | --- |

Between routine appointments, can your patients contact you directly with NF1 concerns?

- Yes
- No

| Page Break |  |
| --- | --- |

How frequently do you make the below assessments for your NF1 patients?

|  | Each visit | Annually | Only if clinically indicated | Not applicable to patient group |
| --- | --- | --- | --- | --- |
| Head circumference |  |  |  |  |
| Height |  |  |  |  |
| Weight |  |  |  |  |
| Blood pressure |  |  |  |  |
| Skin |  |  |  |  |
| Spine / skeletal |  |  |  |  |
| Eyes |  |  |  |  |
| Cardiovascular |  |  |  |  |
| Neurology |  |  |  |  |
| Puberty |  |  |  |  |
| Cognitive and behavioural development |  |  |  |  |

| Page Break |  |
| --- | --- |

Are you aware of the Highly Specialised Complex NF1 Service provided by Manchester University Hospitals NHSFT, and Guy's and St Thomas' NHSFT?

- Yes
- No

Skip To: ophth referral If Are you aware of the Highly Specialised Complex NF1 Service provided by Manchester University Hos... = No

| Page Break |  |
| --- | --- |

Do you know how to contact the Complex NF1 Service for advice or patient referral as needed?

- Yes
- No

| Page Break |  |
| --- | --- |

Are you aware of the eligibility criteria used to refer to the Complex NF1 Service?

- Yes
- No

| Page Break |  |
| --- | --- |

Have you referred any patient to the Complex NF1 Service?

- Yes
- No
- Not applicable to my role

| Page Break |  |
| --- | --- |

Do you refer your NF1 patients for ophthalmology assessments?

- All children, annually until 8 years
- All children, annually until 18 years
- Only if symptomatic
- Not applicable to my role
- I conduct ophthalmology assessments

| Page Break |  |
| --- | --- |

Do you refer your NF1 patients to any of the below? Tick all that apply.

- Child and adolescent mental health
- Adult mental health
- Breast cancer screening (directly or via GP)
- Allied health teams to support development (speech & language, autism support services, etc)
- Genetic counselling (including family planning)
- Occupational therapy
- Physiotherapy
- Specialists as clinically indicated
- Patient support charities (Childhood Tumour Trust, Nerve Tumours UK or Tumour Support Scotland)
- Other (please specify) __________________________________________________

| Page Break |  |
| --- | --- |

To your knowledge, what support is provided for patients when transitioning from paediatric to adult services?

- Transition clinic offered with both paediatric & adult care team in attendance
- Counselled on change in services and new care pathway explained
- Referred to specialist NF1 adult services (transfer of care only)
- Referred to non-NF1 adult services (transfer of care only)
- Discharged to GP (transfer of care only)
- Other (please specify) __________________________________________________

| Page Break |  |
| --- | --- |

Do you feel responsible for coordinating holistic care (including routine monitoring, physical health, mental wellbeing) for your NF1 patients?

- Yes
- No

| Page Break |  |
| --- | --- |

Who do you think **should** be responsible for coordinating holistic care of NF1 **paediatric** **patients**? This includes routine monitoring, physical health, mental wellbeing, and managing the transition to adult services.

- Dermatologist
- General Practitioner
- Geneticist
- Health Visitor
- Neurologist
- Nurse – Community Care
- Nurse – Hospital
- Nurse – Specialist
- Ophthalmologist
- Paediatrician – Community
- Paediatrician – General
- Paediatrician – Specialist
- Paediatric Neurologist
- Other (please specify) __________________________________________________

| Page Break |  |
| --- | --- |

Who do you think **should** be responsible for coordinating holistic care of NF1 **adult** **patients**? This includes routine monitoring, physical health, and mental wellbeing.

- Dermatologist
- General Practitioner
- Geneticist
- Neurologist
- Nurse – Community Care
- Nurse – Hospital
- Nurse – Specialist
- Ophthalmologist
- Other (please specify) __________________________________________________

| Page Break |  |
| --- | --- |

You've selected **Paediatric** **${paed holistic care/ChoiceGroup/SelectedChoicesTextEntry}** and **Adult** **${adult holistic care/ChoiceGroup/SelectedChoicesTextEntry}**.

Do you believe most people within these professions currently have enough knowledge and support (including financial) to provide this NF1 service?

- Yes
- No

| Page Break |  |
| --- | --- |

Display This Question:

If You've selected Paediatric ${q://QID28/ChoiceGroup/SelectedChoicesTextEntry} and Adult ... = No

What additional resources or support do you think are required for a **Paediatric** **${paed holistic care/ChoiceGroup/SelectedChoicesTextEntry}** and **Adult** **${adult holistic care/ChoiceGroup/SelectedChoicesTextEntry}** to coordinate holistic care for NF1 patients?

________________________________________________________________

________________________________________________________________

________________________________________________________________

________________________________________________________________

________________________________________________________________

| Page Break |  |
| --- | --- |

In your opinion, what are the most important management strategies for patients with NF1?

|  | Not important | Somewhat important | Very important |
| --- | --- | --- | --- |
| Educating patients and families about NF1 |  |  |  |
| Diagnosis of learning and behavioural disorders |  |  |  |
| Support for learning and behavioural difficulties |  |  |  |
| Support around emotional wellbeing and mental health |  |  |  |
| Support for education |  |  |  |
| Access to early screening for physical health |  |  |  |
| Direct patient access to NF1 specialists |  |  |  |

| Page Break |  |
| --- | --- |

Please briefly describe how you seek or share NF1 knowledge to collaborate on best practice.

________________________________________________________________

________________________________________________________________

________________________________________________________________

________________________________________________________________

________________________________________________________________

| Page Break |  |
| --- | --- |

| 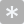 |
| --- |

In your opinion, what are the top 3 research priorities for NF1? The below list was compiled by patients and their families.

- Benefits of full body baseline scan
- Special educational needs
- Correlation with Autism Spectrum Disorder and/or Attention Deficit Hyperactivity Disorder
- Hypermobility
- Pain management
- Management of plexiform neurofibromas
- Relief from itching
- Bowel issues
- Monitoring for malignancy
- Treatment of cutaneous neurofibromas
- Other (please specify) __________________________________________________

End of Block: Question Block

Start of Block: feedback

Please add anything else you’d like to share regarding the current pathway of care for NF1 patients in the UK.

________________________________________________________________

________________________________________________________________

________________________________________________________________

________________________________________________________________

________________________________________________________________

End of Block: feedback
